# cGAS-STING signaling pathway in intestinal homeostasis and diseases

**DOI:** 10.3389/fimmu.2023.1239142

**Published:** 2023-09-14

**Authors:** Yuchen Yang, Li Wang, Ivonne Peugnet-González, Daniela Parada-Venegas, Gerard Dijkstra, Klaas Nico Faber

**Affiliations:** ^1^ Department of Gastroenterology and Hepatology, University of Groningen, University Medical Center Groningen, Groningen, Netherlands; ^2^ Department of Medical Microbiology and Infection Prevention, University of Groningen, University Medical Center Groningen, Groningen, Netherlands

**Keywords:** cGAS, STING, inflammatory bowel diseases, colorectal cancer, intestinal infections, intestinal ischemia/reperfusion

## Abstract

The intestinal mucosa is constantly exposed to commensal microbes, opportunistic pathogens, toxins, luminal components and other environmental stimuli. The intestinal mucosa consists of multiple differentiated cellular and extracellular components that form a critical barrier, but is also equipped for efficient absorption of nutrients. Combination of genetic susceptibility and environmental factors are known as critical components involved in the pathogenesis of intestinal diseases. The innate immune system plays a critical role in the recognition and elimination of potential threats by detecting pathogen-associated molecular patterns (PAMPs) and damage-associated molecular patterns (DAMPs). This host defense is facilitated by pattern recognition receptors (PRRs), in which the cyclic GMP-AMP synthase-stimulator of interferon genes (cGAS-STING) pathway has gained attention due to its role in sensing host and foreign double-stranded DNA (dsDNA) as well as cyclic dinucleotides (CDNs) produced by bacteria. Upon binding with dsDNA, cGAS converts ATP and GTP to cyclic GMP-AMP (cGAMP), which binds to STING and activates TANK binding kinase 1 (TBK1) and interferon regulatory factor 3 (IRF3), inducing type I interferon (IFN) and nuclear factor kappa B (NF-κB)-mediated pro-inflammatory cytokines, which have diverse effects on innate and adaptive immune cells and intestinal epithelial cells (IECs). However, opposite perspectives exist regarding the role of the cGAS-STING pathway in different intestinal diseases. Activation of cGAS-STING signaling is associated with worse clinical outcomes in inflammation-associated diseases, while it also plays a critical role in protection against tumorigenesis and certain infections. Therefore, understanding the context-dependent mechanisms of the cGAS-STING pathway in the physiopathology of the intestinal mucosa is crucial for developing therapeutic strategies targeting the cGAS-STING pathway. This review aims to provide insight into recent findings of the protective and detrimental roles of the cGAS-STING pathway in intestinal diseases.

## Introduction

The intestinal mucosa is a selectively permeable barrier where nutrients are absorbed and immunologic tolerance is induced, but also a physical barrier that prevents against direct tissue injury caused by gut luminal content (1). The intestinal mucosa consists of multiple differentiated cellular and extracellular components: 1) a single-polarized layer of IECs with specialized subtypes (e.g., enterocytes, Paneth cells, goblet cells and enteroendocrine cells) attached to each other and to basal membrane; 2) cells within the subepithelial lamina propria (such as stromal cells and immune cells); 3) biochemical elements secreted by those cells (e.g. regenerating islet-derived protein 3 gamma (Reg3γ) and defensins) (2). Combinations of genetic susceptibility and environmental and lifestyle factors, including dietary and microbiome changes, alcohol consumption, exposure to toxins or detergents, infections and smoking, contribute to intestinal barrier dysfunction, mucosal inflammation and dysbiosis. These factors are increasingly acknowledged as critical mechanisms involved in the development of intestinal diseases (3).

As the first line of defense against complex exposures and a bridge to the adaptive system, the innate immune system has evolved various sensing mechanisms modulated by PRRs to detect and counteract microbial invasion by recognizing PAMPs and DAMPs (4). PRRs mainly include Toll-like receptors (TLRs) (5), retinoic acid-inducible gene I (RIG-I)-like receptors (RLRs) (6), nucleotide oligomerization domain (NOD)-like receptors (NLRs) (7), C-type lectin receptors (CLRs) (8), absent in melanoma-2 (AIM2)-like receptors (ALRs) (9), as well as other DNA and RNA sensors (10–15). The competence of sensing microbial pathogens through recognition of their nucleic acids (RNA and DNA) has been considered as a key feature of innate immunity in mammalian cells (16, 17). One major family of cytokines fundamentally involved in antimicrobial host defense are the type I IFN, which exerts antiviral, anti-proliferative, anti-tumor and a multitude of regulatory actions on innate and adaptive immune responses, involving antigen presentation and the differentiation of both CD4^+^ and CD8^+^ T cells (18, 19). Just over a decade ago, a signaling pathway that controls the production of type I IFN was discovered, which consists of the cGAS for generating the second messenger cGAMP and cGAMP receptor STING (20, 21). The nucleic acids of bacteria, viruses and parasites can enter cells through various mechanisms, such as phagocytosis, endocytosis and membrane fusion (22–24). Pathogen-related infections also causes injury to host cell and release of their genomic and mitochondrial DNA (mtDNA) (25–27). Upon direct binding with host or foreign dsDNA, cytosolic cGAS undergoes a switch-like conformational change that activates its enzymatic activity, converting GTP and ATP to the second messenger cGAMP (28, 29). Subsequently, cGAMP binds to STING, a transmembrane adaptor located at the endoplasmic reticulum (ER), to promote the phosphorylation of TBK1 and IRF3, and thereby inducing type I IFN production and NF-κB-dependent expression of pro-inflammatory cytokines in the innate immune system (30). In addition to cGAMP synthesized by cGAS, the bacterial-produced CDNs (c-di-AMP and c-di-GMP) and canonical cGAMP (e.g. 3’3’-cGAMP) can be directly sensed by STING as well, which is reviewed in detail by Liu et al. (31). Multiple studies have demonstrated the critical role of the cGAS-STING signaling in intestinal pathophysiology, however, both protective and detrimental roles of cGAS-STING signaling have been reported. On the one hand, the activation of cGAS-STING signaling is associated with the severity of intestinal inflammation. On the other hand, it plays a critical role in maintaining the intestinal structure and environment, as well as protecting against tumorigenesis and infections. It is known that inflammation displays opposing effects in cancer development and immunotherapy, such as immune checkpoint inhibitors and cancer vaccines. Inflammation can facilitate tumorigenesis and immune resistance but can also induce differentiation of immune cells and antigen presentation to perform an anti-cancer immune response (32–34). Therefore, it is necessary to elucidate the shift from a promoter to a protector role of the cGAS-STING pathway in the transition from intestinal inflammation, cancer, infections and tissue repair. Understanding the intricate mechanisms of the cGAS-STING pathway in the pathophysiology of the intestinal mucosa is crucial for the development of effective therapeutic interventions targeting this signaling pathway. In this review, we describe recent findings of the cGAS-STING pathway according to its protective and detrimental roles in intestinal homeostasis and diseases.

## The cGAS-STING signaling pathway

**Figure 1:**
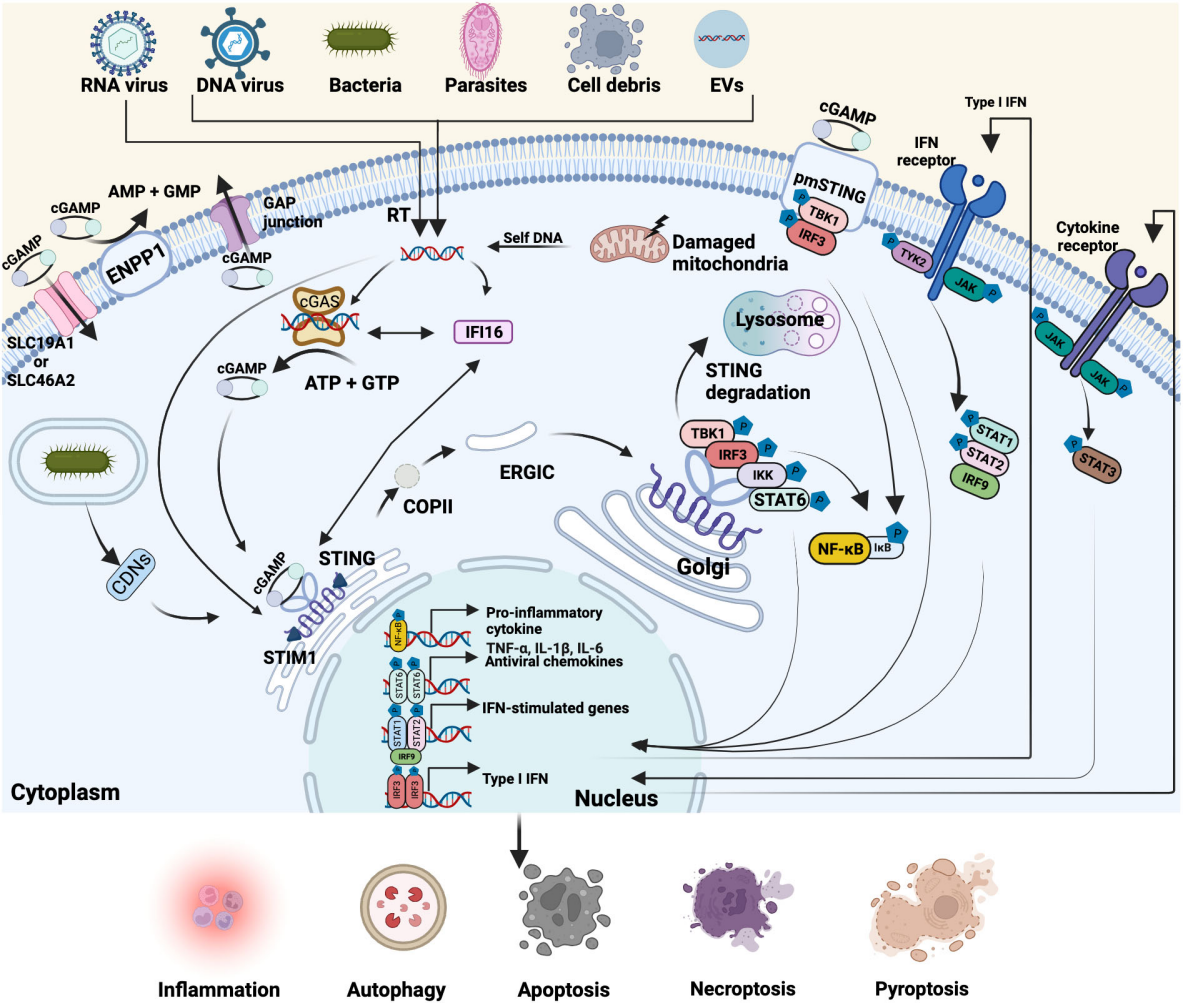
Overview of the cGAS-STING pathway. Cyclic GMP-AMP synthase (cGAS)-stimulator of interferon genes (STING) pathway is involved in multiple biological processes. Cytoplasmic cGAS can recognize exogenous and endogenous double-strand DNA (dsDNA) from various sources, including DNA viruses, reverse-transcription of RNA viruses, bacteria, parasites, cell debris, extracellular vesicles (EVs), as well as the damaged mitochondria- and self-DNA from the nucleus. Interferon-gamma inducible protein 16 (IFI16) directly interacts with cytosolic dsDNA, cGAS and STING, facilitating the activation of cGAS and STING. Activated cGAS then catalyzes the synthesis of the second messenger cyclic GMP-AMP (cGAMP) from ATP and GTP. Cyclic dinucleotides (CDNs) of bacteria, cGAMP or dsDNA binds to endoplasmic reticulum (ER)-located STING that is retained by stromal interaction molecule 1 (STIM1) and initiates STING translocation to the Golgi apparatus *via* coat protein complex II (COPII) and ER-Golgi intermediate compartment (ERGIC). Then STING recruits and phosphorylates TBK1 and IRF3 and simultaneously activates IΚB kinase (IKK) and signal transducer and activator of transcription 6 (STAT6), triggering the transcription of pro-inflammatory cytokines, antiviral chemokines, IFN-stimulated genes and Type I IFN. In parallel, some cGAMP can be delivered to the outside of cells through the GAP junction, binding with the plasmatic membrane (pmSTING) and driving the same activation response or enter other cells through solute carrier family 19 member 1 (SLC19A1) and SLC46A2. The rest of cGAMP can be degraded by membrane hydrolase ectonucleotide pyrophosphatase phosphodiesterase 1 (ENPP1). Post-Golgi STING vesicles are either fused or encapsulated by lysosomes and gradually eliminated. After type I IFN and cytokines (e.g. IL-6) are secreted outside, they can bind to IFN receptor and cytokine receptor (e.g. IL-6R/GP130) on the membrane respectively and then activate Janus kinases (JAK)-STAT signaling pathway. Created with BioRender.com.

The cGAS-STING signaling pathway is graphically presented in **Figure 1**. The *MB21D1* gene (also known as C6orf150), located on chromosome 6q13, is one of the most rapidly diverging genes in the human genome. It encodes the 60 kDa nucleotidyltransferase enzyme cGAS of 522 amino acids and is activated by endogenous dsDNA (e.g., damaged genomic DNA and mtDNA) and exogenous dsDNA (from viruses, bacteria and parasites) (35, 36). cGAS belongs to the structurally conserved cGAS/DncV-like nucleotidyltransferase superfamily, which consists of a positively charged N-terminal domain (aa1-160) and conserved C-terminal domain (aa161-522) that includes the NTase core (aa160-330) and the male abnormal 21 (Mab21) domain (aa213-513) with zinc-ribbon insertion (37–39). Human cGAS has three DNA-binding sites, defined as site A, B and C, where cGAS interacts with dsDNA *via* electrostatic interactions and hydrogen bonds. The interface of cGAS and dsDNA form salt bridges with the phosphate backbone of the DNA and then form a 2:2 complex with dsDNA in an asymmetrical manner (29, 40). The binding of cGAS to DNA depends on the DNA length in a sequence-independent manner (41, 42). Human cGAS K187 and L195 are two substitutions in the cGAS DNA-binding surface that enhance the ability of cGAS to distinguish short (~20 bp) and long (>45 bp) DNAs (40). The activity of cGAS is dependent on the length of the dsDNA and some species-specific differences have been reported (29, 43). While the catalytic activity of mouse cGAS activity is strongly induced by short dsDNA of ~18 bp (29), long dsDNA (>45 bp) more efficiently promotes human cGAS enzymatic activity than the shorter counterparts. Enzymatic activity of human cGAS appeared also dependent on the concentrations of zinc (43). The C-terminal domain of cGAS is critical for activating the enzymatic activity upon dsDNA binding through triggering conformational changes (42, 44, 45), in which the conversion of ATP and GTP to cGAMP happens (21, 44, 46). Under physiological conditions, the continuous cytosolic clearance of endogenous DNA *via* cytosolic 3-prime repair exonuclease 1 (TREX1) and lysosomal DNase II is important to avoid the overactivation of the cGAS-STING signaling (47, 48). Notably, other DNA sensors, such as IFI16, DDX41, DAI (DLM-1/ZBP1) and MRE11 might also be involved in the modulation of the cGAS-STING-type I IFN pathway (10–13). For instance, IFN-γ inducible protein 16 (IFI16) can bind to exogenous DNA and form a complex with cGAS, synergizing cGAMP-induced phosphorylation and translocation of STING (13). Detailed analysis interaction between these other DNA sensors and the cGAS-STING signaling pathway is needed to better understand the context-specific role of cGAS-STING activation.

STING (also named MITA, MPYS and ERIS), encoded by the *TMEM173* gene, is an evolutionary conserved transmembrane protein consisting of 379 amino acids with a molecular weight of 42 kDa, widely existing in diverse species and cell lineages, including endothelial cells, epithelial cells, immune cells (T cells, macrophages, dendritic cells (DCs)) and hematopoietic cells (20, 49, 50). STING predominantly resides in the ER and forms a highly structured wing-like hydrophobic dimer facing toward the cytosol (51). The N-terminal domain of STING includes four transmembrane helices to anchor this adaptor protein to the ER membrane, and the C-terminal domain of STING comprises a ligand-binding domain and a C-terminal tail, which are responsible for binding cGAMP, CDNs and TBK1 (52–54). Recently, plasmatic membrane STING (pmSTING), an alternatively-spliced STING isoform, located in the cytoplasmic membrane with C-terminal tail projecting outside the cell, has been identified in many species, including humans. Human pmSTING can sense extracellular cGAMP and activates TBK1-IRF3-type I IFN signaling the same way as the canonical one (55). Since the subcellular localization of STING can influence its function and interaction with cellular and extracellular components, further studies on the functional differences of ER-located STING and pmSTING are needed. During the resting/non-activated state, STING is retained in the ER by associating with stromal interaction molecule 1 (STIM1), which acts as an ER retention factor to suppress the activity of STING (56). Viral infection begins with the attachment of the virus to the surface of host cells mediated by viral entry proteins and host surface receptors (e.g. proteins, carbohydrates or lipids). Once attached, viruses can enter cells and release genetic material by direct fusion of the viral envelope with the membrane of host cells or endocytosis (57). Also, bacteria and parasites possess various exquisite tools for cell adhesion and host cell invasion (58, 59). Upon binding with dsDNA from pathogens or damaged cells, cytoplasmic cGAS undergoes a conformational change and forms a sandwiched-like structure with dsDNA (28, 60), which induces liquid-liquid phase separation and the formation of liquid-like droplets that function as micro-reactors to enhance cGAMP production by concentrating enzymes and reactants, which promotes the conversion of ATP and GTP to cGAMP (21, 43, 44). The binding of cGAMP to STING leads to a conformational change of the ligand-binding domain of STING, triggering a release of the C-terminal domain of STING, and subsequently, a disulfide-stabilized polymer is formed for interacting with TBK1 and IRF3 (20, 54, 61). The cGAMP-mediated disruption of the interaction between STING and ER-resident protein STIM1 initiates the intracellular trafficking of STING from ER to the Golgi apparatus through the coat protein complex II (COP-II)-vesicles and ER-Golgi intermediate compartment (ERGIC) (31). Meanwhile, many positive regulators of STING trafficking from the ER to Golgi are involved in this process, which have been extensively reviewed by Taguchi et al. (62). For example, STING ER exit protein (STEEP) interacts with STING and promotes trafficking from the ER to Golgi through promoting the accumulation of phosphatidylinositol-3-phosphate (PI3P) and ER membrane curvature (63). The palmitoylation of STING at cysteines 88 and 91 is essential for the location of STING in the Golgi and the initiation of downstream signal cascades (54, 64). In addition to recognizing cGAMP produced by cGAS, STING also binds dsDNA and c-di-GMP directly (61, 65), while the direct binding of c-di-AMP to STING remains unclear so far. Then STING oligomers recruit TBK1 *via* its highly conserved PLPLRT/SD motif in the C-terminal tail and induce clustering and trans-autophosphorylation of TBK1 (66, 67). Activated TBK1 phosphorylates STING at the C-terminal *p*L*x*IS motif (*p*, hydrophilic residue; *x*, any residue; S, phosphorylation site), which allows STING to recruit IRF3 and subsequently binds to the conserved, positively charged surfaces of IRF3, thereby promoting its phosphorylation and activation by TBK1. The phosphorylated IRF3 dissociates from STING, undergoes dimerization, and finally translocates to the nucleus (67, 68). In the nucleus, the phosphorylated IRF3 homodimers bind to DNA at the interferon-stimulated response elements (ISREs) and orchestrate the expression of type I IFN (69). Eventually, when type I IFN are secreted outside the cells, they can be recognized by their receptors. This activates Janus kinases (JAKs) and tyrosine kinase 2 (TYK2), followed by the phosphorylation of signal transducer and activator of transcription protein 1 (STAT1), STAT2 and IRF9, which drives the production of interferon-stimulated genes (ISGs) to establish antipathogenic immunity functions *via* JAK-STAT signaling pathways (70). In addition, STING may also activate STAT6 independent of JAKs through phosphorylating its Ser^407^ *via* TBK1 (71). Apart from eliciting type I IFN response, STING activation can also trigger NF-κB signaling (72). Recent evidence indicated that TBK1/IKKϵ redundantly induces phosphorylation of STING and promotes the recruitment and phosphorylation of transforming growth factor-β-activated kinase 1 (TAK1) and inhibitor of NF-κB kinase subunit beta (IKKβ). This leads to the phosphorylation and degradation of IκB inhibitory proteins, which further promotes the translocation of NF-κB (p65 and p50) into the nucleus and triggers NF-κB signaling activation (73, 74). Interestingly, compared with human and mouse STING, zebrafish STING contains a unique C-terminal tail module that is responsible for the enhanced NF-κB activation. Further investigation showed that zebrafish STING sequence contains the closest homology with TRAF6 recruitment motifs, which is indispensable for zebrafish STING-induced NF-κB signaling activation, but not for human and mouse, indicating there might be some species-specific differences in cGAS-STING-induced NF-κB activation (73, 75). Moreover, the activation of the cGAS-STING pathway facilitates non-canonical NF-κB signaling by recruiting mitogen-activated protein kinase kinase kinase 14 (MAP3K14/NIK) and NIK-STING interaction might act to synergize STING-dependent type I IFN response (76–78). Those activated molecules induce a set of cytokines, chemokines and immunomodulatory genes that are responsible for inflammatory or immune responses, ultimately establishing an antipathogen state (79). For instance, after IL-6 are secreted outside, they bind to their cytokine receptors (IL-6R/GP130) on the membrane of cells, leading to the homodimer formation of STAT3, which in turn triggers target gene expression to influence cell growth and survival (80, 81). As canonical NF-κB activation has been reported to enhance STING signaling through blocking STING degradation by regulating microtubule-mediated STING trafficking from the Golgi apparatus to lysosomes (82), and activation of non-canonical NF-κB pathway resulted in decreased type I IFN expression (83), suggesting canonical and non-canonical NF-κB pathway might play a different role in modulating STING signaling. Interestingly, cGAMP is synthesized in the cytosol and cannot cross the cell membrane due to its two negative charges. However, it was recently shown that cGAMP can be exported to the extracellular space through gap junctions (84), exosomes (85), LRRC8 (86), multidrug-resistance-associated protein 1 (MRP1) (87) and recognized by pmSTING to signal other types of cells in a paracrine manner. With the help of cGAMP importers SLC19A1 and SLC46A2, it is likely that extracellular cGAMP can also be detected by ER-located STING (88, 89). Extracellular cGAMP can also be degraded by the membrane hydrolase ectonucleotide pyrophosphatase phosphodiesterase 1 (ENPP1) that has a catalytically active domain toward extracellular through hydrolyzing the 2’,5’-phosphodiester linkage and 3’,5’-phosphodiester linkage of cGAMP (90, 91). In ENPP1 knockout 293T cells, a significant increase of extracellular cGAMP molecules was detected (92), demonstrating the potential of ENPP1 in regulating the cGAS-STING signaling. After fulfilling its role, post-Golgi STING vesicles are either fused or encapsulated by lysosomes and gradually eliminated (93).

## The cGAS-STING pathway in intestinal homeostasis

**Figure 2:**
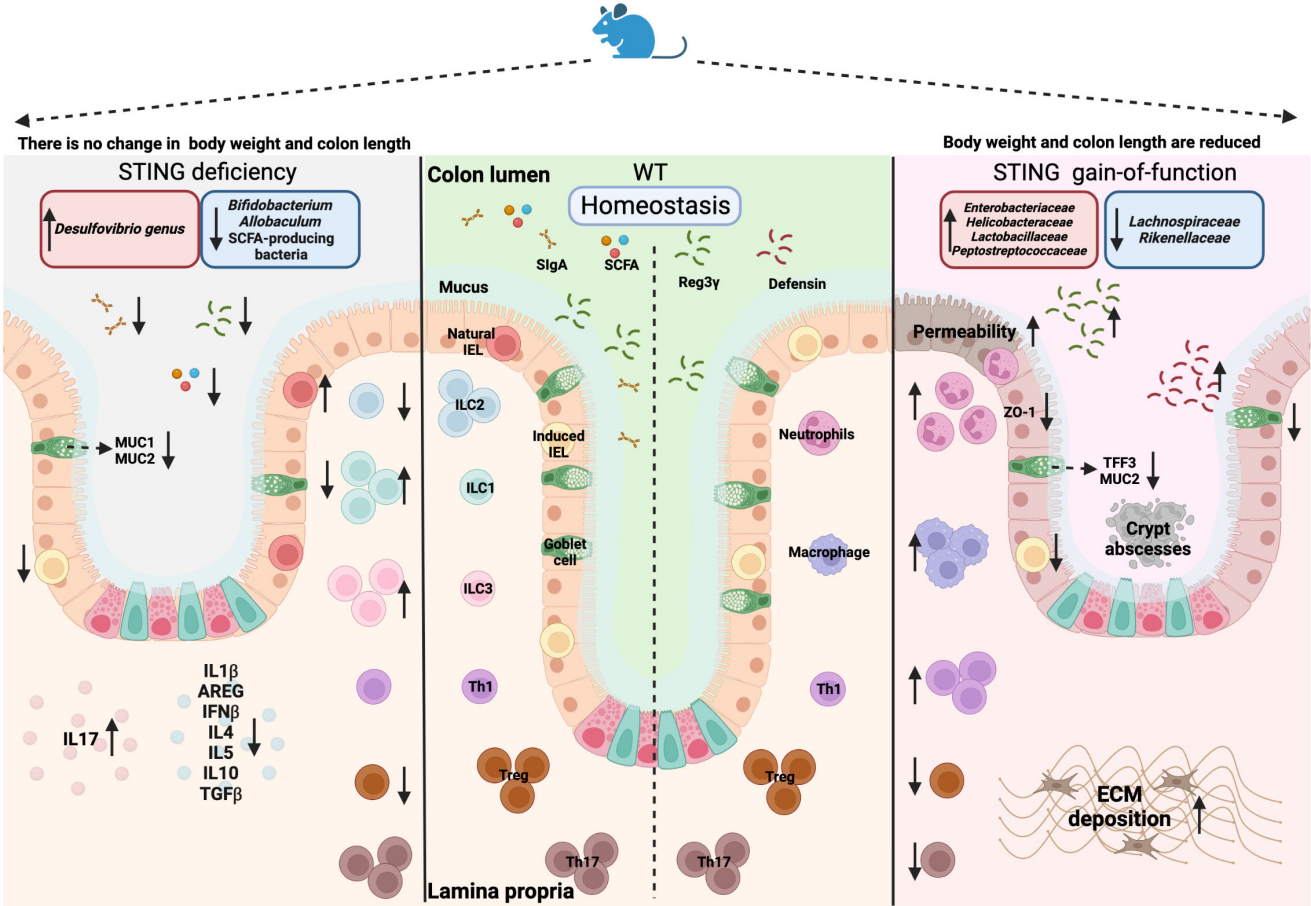
Overview of the STING deficiency (*Sting* ^-/-^) and STING gain-of-function (*Sting* ^+/N153s^) on mice models. Created with BioRender.com.

**Figure 3:**
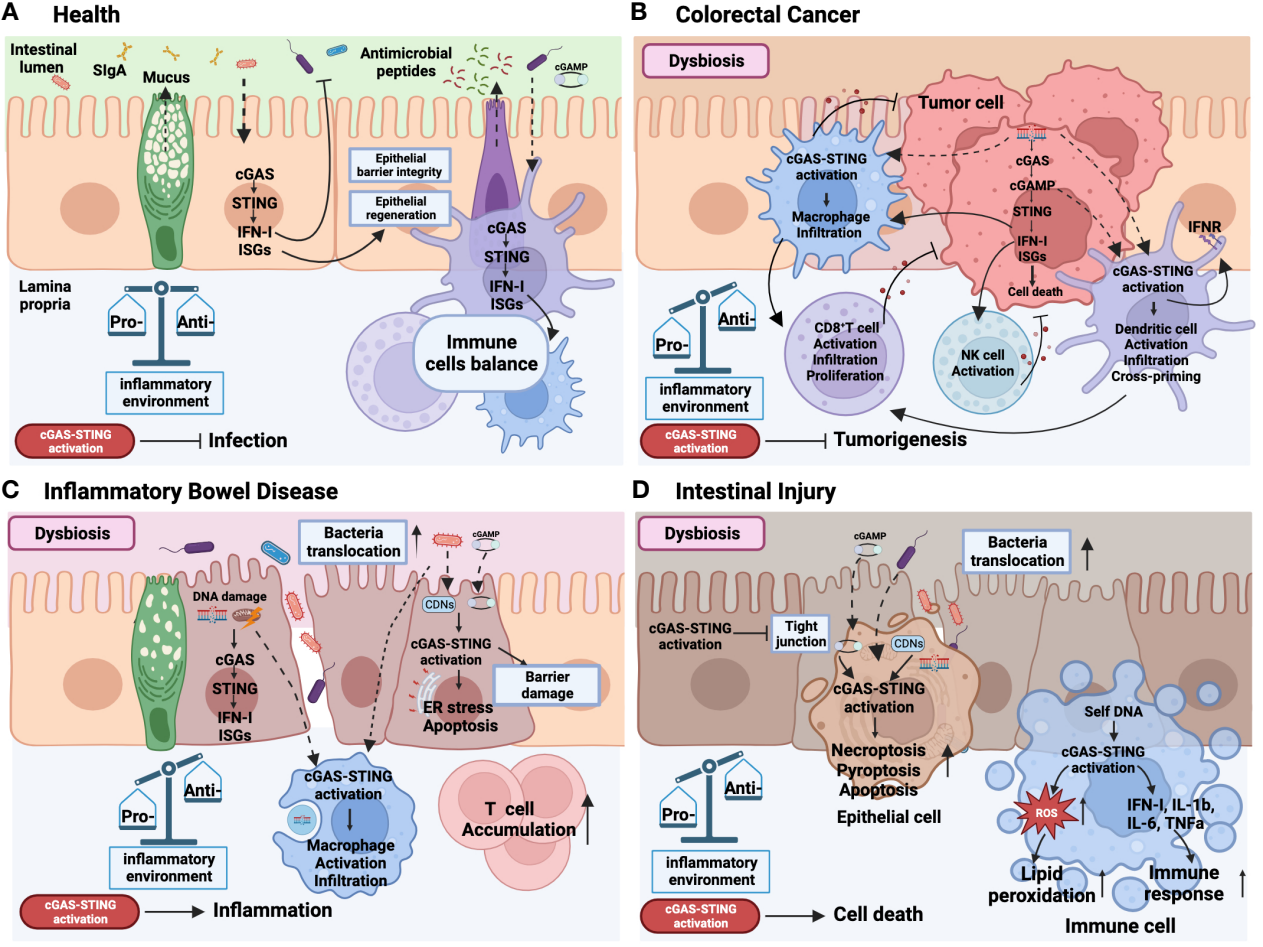
Role of cGAS-STING signaling activation in health and intestinal diseases. In the context of health **(A)**, the intricate symbiotic interplay between the intestinal microbiota and the colonic mucosa relies on the intrinsic STING pathway present in both epithelial and immune cells. This dynamic interaction facilitates the defense against pathogenic infections by stimulating the secretion of type I interferons (IFN-I) and Interferon-stimulated gene (ISGs), reinforcing the integrity and regeneration of the epithelial barrier, promoting the production of antimicrobial peptides by Paneth cells, mucus synthesis by goblet cells, secretory IgA (SIgA) expression by plasma cells. This mutually beneficial relationship is crucial for maintaining immune cell homeostasis and establishing a harmonious and balanced gut environment. Colorectal cancer **(B)** is marked by an imbalanced state of the microbiota. Within this context, the release of DNA from dead tumor cells triggers the activation of the STING pathway in tumor cells and immune cells. This dual activation cascade promotes a pro-inflammatory immune response within the tumor microenvironment, effectively impeding tumorigenesis. Notably, STING activation facilitates the infiltration, activation, proliferation, and cross-priming of dendritic cells, macrophages, CD8^+^ T cells, and NK cells. Inflammatory bowel disease (IBD) **(C)** also manifests dysbiosis and the translocation of pathogenic microorganisms into the lamina propria (LP) due to a compromised epithelial barrier. Bacterial DNA or cyclic dinucleotides (CDNs) and self-DNA derived from damaged cells, including double-stranded DNA (dsDNA) and mitochondrial DNA (mtDNA), induce the activation of STING in both epithelial and immune cells, thereby exacerbating intestinal mucosal inflammation. Furthermore, STING activation and its downstream effectors elicit endoplasmic reticulum (ER) stress, and apoptosis, contributing to epithelial barrier damage. Consequently, macrophage infiltration and activation, along with T cell accumulation in the LP, are promoted by STING activation, perpetuating the imbalance of pro-inflammatory cells and cytokines in IBD. Similar to IBD, intestinal injury **(D)** is characterized by dysbiosis, epithelial damage, and the translocation of bacteria. Bacterial DNA or CDNs and self-DNA from damaged or dead cells play a significant role in activating the STING pathway, leading to cellular death, inflammation, and intestinal barrier disruption. Furthermore, activation of the STING pathway in immune cells elevates reactive oxygen species (ROS) levels, subsequently causing lipid peroxidation and triggering the production of IFN-I and pro-inflammatory cytokines. This collective response perpetuates a state of sustained inflammation within the intestinal environment. The solid line represents effects, the dotted line represents translocation. Created with BioRender.com.

The intestinal mucosa is exposed to commensal microbiota and potential pathogenic microbiota, their metabolites and a multitude of ingested luminal factors. The intestinal epithelial barrier is the first line defense against gut microbes, which is composed of stem cells, differentiated epithelial cells and their secreted products (e.g., mucins produced by the goblet cells and antimicrobial peptides produced by Paneth cells), and immune cells (e.g., intraepithelial lymphocytes (IELs)) (94). Additionally, cytokines and chemokines released by immune cells in the lamina propria (LP), such as mononuclear phagocytes and antigen-presenting cells (e.g., macrophages and DCs), as well as antibodies (e.g., Immunoglobulin A (IgA)) produced by plasma cells, protect the host against infections and harmful antigens (95). The antipathogenic response by epithelial and immune cells is mediated by the recognition PAMPs or DAMPs (e.g., lipopolysaccharides (LPS) and dsDNA) through diverse PRRs (96). From the various PRRs, increasing evidence shows that STING plays a central role in modulating and maintaining intestinal homeostasis (97–100). Whole-body STING knockout (*Sting* ^-/-^) mice displayed alterations in the mucosa and intestinal microbiota when compared to WT mice, while no obvious weight loss and length of the colon were observed **(**left panel in **Figure 2** **)**. Elongated villi in the small intestine and shorter crypts in the colon were observed. In the colon, there were also decreases in induced IELs (expressing TCRαβ), goblet cells, mucin production (MUC-1 and MUC-2) and secreted IgA (sIgA). In the lamina propria, there were elevations in innate lymphoid cells (ILC) 1 and ILC3, as well as reductions in ILC2 and anti-inflammatory T regulatory (Treg) cells. IL17 secretion, which is commonly associated with an inflammatory response, was upregulated, and accompanied by a decrease in cytokines associated with tissue regeneration and anti-inflammatory function (101). With regard to microbiota alterations, a reduction in *Bifidobacterium* (Actinobacteria phylum) and *Allobaculum* (Firmicutes phylum) and an increase in *Desulfovibrio genus* (Proteobacteria phylum) were observed in *Sting* ^-/-^ mice, suggesting a shift to a pro-inflammatory microbiota profile (101). Furthermore, a decrease in serum and fecal IgA, short-chain fatty acid (SCFA)-producing bacteria and SCFA fermentation were observed in *Sting* ^-/-^ mice (102). STING also enhanced the production of the antimicrobial peptide Reg3γ in a type I IFN-dependent manner, which could limit damage to intestinal tissues by sustaining a protective shield against bacterial colonization and translocation (102). In addition to *Sting^-/-^* mice, also mice with constitutive-active STING have been generated and analyzed for intestinal functions. STING gain-of-function *Sting* ^+/N153s^ (N153S) mice presented weight loss and reduction of colon length compared to WT mice, together with loss of intestinal barrier integrity as shown by higher intestinal permeability, lower level of ZO-1, loss of goblet cells along with their products (MUC2 and TFF3), as well as a decrease in induced IELs along with increased defensin and Reg3γ production **(**right panel in **Figure 2**). STING gain function caused a decrease in Treg cells and Th17 in the lamina propria, but an increase in other pro-inflammatory cells, such as neutrophils, macrophages and Th1 cells. Notably, N153S mice displayed excessive extracellular matrix (ECM) deposition in the colon (103). The changes in the number of stem cells, Paneth cells and enteroendocrine cells have not been described in either of the 2 mouse models, which can be interesting perspectives in exploring the function of STING in the epithelial cells. N153S mice also showed altered gut microbiota composition compared to WT mice, displaying an increase in the bacterial families *Enterobacteriaceae*, *Helicobacteraceae*, *Lactobacillaceae*, *Peptostreptococcaceae* and a decrease in *Lachnospiraceae* and *Rikenellaceae* (103). In addition, the cGAS-STING-type I IFN axis promoted intestinal regeneration after irradiation injury (104, 105). These studies indicate that intrinsic STING signaling is important to maintain mucosal homeostasis and adequate symbiosis with gut microbiota **(** **Figure 3A** **)**.

## The cGAS-STING pathway in colorectal cancer

Colorectal cancer (CRC) is the third most common cancer worldwide associated with high mortality. Although treatment of CRC has slowly but steadily improved in many countries, its 5-year survival rate is still less than 50% in low-income countries. The biological heterogeneity and complex tumor microenvironment of CRC impede the development of efficient and personalized therapeutic strategies (106). Recently, several studies have shown that the cGAS-STING signaling is critical to protect against tumorigenesis **(** **Figure 3B** **)** and preserve the efficacy of anti-tumor therapies such as immune checkpoint therapy, chemotherapy and radiotherapy (**Table 1**). Many colon adenocarcinoma cell lines show poor response to dsDNA-induced activation of the STING pathway and type I IFN production. Epigenetic silencing of STING and cGAS inhibited tumor cell death and anti-tumor response (126). STING knockout CT26 colon carcinoma cells significantly promoted tumor growth in immune-competent C57BL/6 mice and BALB/c mice compared to parent CT26 cells (122). CRC patients with high STING expression exhibited increased intratumoral CD8^+^ T cell infiltration and less frequent lymphovascular invasion in the early stage of cancer and had longer overall and recurrence-free survival compared to CRC patients with low STING expression (124), which has also been confirmed by Yang et al. (116). These findings suggest that the level of STING can be used as a clinical predictor for CRC progression. Further research indicated that the growth of MC38 tumors can be effectively suppressed by intratumoral treatment with the STING agonist 3’3’-cGAMP leading to enhanced intratumoral infiltration and activation of CD8^+^ T cells (124), suggesting that the ability of STING to suppress tumor growth may rely on regulating immune cells and the activation of STING could be a therapeutic strategy for CRC treatment. The anti-tumor role of STING has also been observed in the mouse model of azoxymethane/dextran sodium sulfate (AOM/DSS)-induced colitis-associated cancer (CAC) (108–110). *Sting^gt^* is an I199N missense mutant allele leading to inactive STING. Homozygous *Sting* ^gt/gt^ mice do not produce IFN-β in response to cyclic dinucleotides or *Listeria monocytogenes* infection (127). *Sting^gt/gt^* mice housed in a specific pathogen-free (SPF) facility are highly susceptible to AOM/DSS-induced CAC. These mice demonstrated more severe colonic hyperplasia during the early stages of tumorigenesis and failed to restrict activation of the NF-kB- and STAT3-signaling pathways and expressed increased levels of the pro-inflammatory cytokines IL-6 and KC (a chemokine that is capable of recruiting neutrophils) (109). Exacerbating CAC has been also reported in AOM/DSS-induced *Sting* ^-/-^ mice as well (108). The protective effect of STING was also observed in AOM/DSS-induced *Sting* ^-/-^ mice housed in the environment containing *Helicobacter* spp. (*Helico*) that is known to influence the outcome of colitis (110). Regarding that AOM/DSS-induced *Sting* ^-/-^ mice housed in *Helico*-SPF barrier failed to produce IL-10 compared with WT mice, STING may also interact with opportunistic pathogens to produce anti-inflammatory cytokines for maintaining gut immune homeostasis (110). However, the role of cGAS in tumorigenesis is controversial. AOM/DSS-treated *Cgas* ^-/-^ mice showed no obvious increase in polyp formation compared to WT mice regardless of the *Helico* status in their study (110), whereas in another study, *Cgas* ^-/-^ mice displayed higher mortality upon AOM/DSS treatment compared with *Sting^gt/gt^* and interferon alpha and beta receptor (*Ifnar*)^-/-^ mice (107). Since the above studies used different drug delivery strategies and different mice lineages, the discrepancies between these studies are not clarified yet. Thus, more studies are needed to investigate the difference and association of cGAS, STING and IFNAR in the progression of CRC.

**Table 1:** Strategies targeting the cGAS-STING pathway in colon cancers.

Cancer type	Mice models	GM-mice lineages	Human Cell lines	Human clinical data	Main finding about the mechanisms and role of the cGAS-STING signaling and/or possible therapies/agents targeting the cGAS-STING pathway to improve disease progression/outcomes	Reference
**CAC**	AOM/DSS-induced CAC	*Cgas^-/-^*	-	-	**cGAS restricted tumorigenesis in both hematopoietic and nonhematopoietic cells.** cGAS deficiency promoted the proliferation of IECs and caused mucin depletion during cancer development. cGAS deficiency enhanced the activation of STAT3, T cells exhaustion, myeloid-derived suppressor cells and Th17 cells accumulation in the colon. Intraperitoneal injection of **cGAMP** partially reduced the number of tumors.	(107)
**CAC**	AOM/DSS-induced CAC	*Sting^-/-^*	HT-29	CAC tissues (n = 5-7)	**STING had a protective role in the tumorigenesis of CAC.** STING was elevated in the colon tissue of CAC patients and protected tumorigenesis by enhancing Syk-mediated pyroptosis of tumor cells. Intraperitoneal injection of STING agonist **DMXAA** stimulated p-Syk and cleaved GSDMD expression, reduced structural heteromorphism, the number of adenocarcinomas, and increased immune cell infiltration. STING agonist **cGAMP** reduced cell viability and induced pyroptosis of HT-29 cells through Syk signaling.	(108)
**CAC**	AOM/DSS-induced CAC	*Sting^gt/gt^*	-	-	**The important role for STING in mediating protection against colorectal tumorigenesis.** *Sting^gt/gt^* mice showed an increase in the number of tumors, histological score and dysplasia of colon. STING deficiency failed to restrict NF-kB- and STAT3-signaling mediated colon inflammation and tumorigenesis.	(109)
**CAC**	AOM/DSS-induced CAC	*Sting^-/-^*	–	–	**STING might interact with gut bacteria in controlling gut immune homeostasis**. *Sting^-/-^* mice housed in *Helicobacter* spp.-SPF showed a lower survival rate, reduced IL-10, and higher colon inflammation upon AOM/DSS treatment. AOM/DSS-induced *Sting* ^-/-^ mice housed in the environment containing *Helicobacter spp.* had a higher number of polyps, which can be ameliorated by antibiotics treatment.	(110)
**CRC**	CT26 tumor-bearing model	-	HCT116 HT-29 SW620	-	**The combination of lovastatin and chemotherapy is a promising therapeutic strategy for colon cancer.** DNA damage induced by chemotherapeutic drugs could activate STING pathway. SHP2 inhibition in colon cancer cells reduces the activation of STING pathway. SHP2 agonist **lovastatin** impaired DNA repair *via* the dephosphorylation of PARP1 and therefore increased acumination of dsDNA and subsequently enhanced STING pathway-medicated anti-tumor immunity.	(111)
**CRC**	MC38 and CT26 tumor-bearing model	*Sting^-/-^* *Cgas* *Ifnar^-/-^*	–	STING-High, n = 43, STING-Low, n = 15	**STING pathway was involved in 5‐Fluorouracil-triggered cancer-cell-initiated anti-tumor immunity.** Higher STING expression was associated with better chemotherapy response and survival in human colon cancer. Chemotherapeutic drug **5‐Fluorouracil** reduced tumor burden was dependent on cancer-cell-intrinsic cGAS-STING-IFN signaling. IFN sensed by bone-marrow-derived cells and the elevated anti-tumor T cells were critical to suppress tumor growth.	(112)
**CRC**	CT26 tumor-bearing model	-	HCT116 SW480	-	**Midostaurin may have the potential to enhance immunotherapy in clinical practice by activating STING pathway.** **Midostaurin** inhibited the growth of colorectal adenocarcinoma cells in a dose- and time-dependent way, which was associated with the formation of cytosolic DNA and activation of the cGAS-STING pathway. Oral administration **midostaurin** combined with anti-PD-1 enhanced anti-PD-1 efficacy by increasing STING and IFNβ expression of the tumors.	(113)
**CRC**	CT26 tumor-bearing mice model.	*Sting^-/-^* *Ifnar^flox/flox^* *Cd11c* ^Cre^ *Rag1* ^-/-^	–	–	**Bifidobacterium showed promise in enhancing local anti-CD47 immunotherapy through STING signaling.** Intratumoral/intravenous/oral administration of ***Bifidobacterium*** facilitated local anti-CD47 immunotherapy in a STING-IFN-dependent and T cell-dependent manner. Intratumoral injection of **DMXAA** similarly improved the anti-tumor efficacy of anti-CD47 therapy in nonresponder mice compared to *Bifidobacterium*.	(114)
**CRC**	MC38 tumor-bearing model	*Cgas* ^–/–^, *Sting* ^–/–^, *Rag1* ^–/–^ *Ifnar* ^–/–^	-	-	***Lactobacillus rhamnosus* GG (LGG) improving immune checkpoint blockade was associated with STING pathway.** Oral administration of **LGG** enhanced the anti-tumoral effect of PD-1 immunotherapy by increasing tumor-infiltrating DCs and T cells through the cGAS-STING pathway-mediated IFN production.	(115)
**CRC**	CT26 tumor-bearing mice model.	*-*	–	CRC tissues (n = 160)	**Intratumoral STING activation normalized tumor vasculature and the tumor microenvironment.** High endothelial STING expression was correlated with higher CD8^+^ T cells infiltration and less lymphovascular invasion within colorectal tumor tissues. Intratumoral injection of the STING agonist **RR-CDA** (also called **MIW815** or **ADU-S100**) combined with VEGRF2 blockade induced tumor regression by enhancing CD8^+^ T cell infiltration.	(116)
**CRC**	MC38 tumor-bearing model	*Senp3* ^flox/flox^, *Cd11c^Cre^* *Cgas* ^-/-^ *Sting* ^-/-^	HEK293T	CRC tissues (n = 28)	**Oxidative stress promoted STING-mediated DCs anti-tumor immune responses.** DCs from CRC displayed elevated SENP-IFI16 interaction and decreased IFI16 SUMOylation. DNA-induced interaction between IFI16 and STING was correlated with the increased of p-STING and p-TBK1 in DCs from patients CRC tissues. SENP3 sensed oxidative stress to promote anti-tumor response of DCs in a STING-dependent way.	(117)
**CRC**	MC38-OVA tumor-bearing model	*Sting* ^-/-^	–	–	**RT/anti-SIRPα/anti-PD-1 promoted anti-tumor efficiency in a STING-dependent way.** RT-induced CD47 and PD-L1 upregulation restrained radiation-induced immune priming in CRC cells. Host STING activation is critical for TAA-specific CD8^+^ T cross-priming, APC mobilization and myeloid compartment activation upon RT/anti-SIRPα/anti-PD-1 therapy.	(118)
**CRC**	CT26 tumor-bearing mice model.	-	Colonoids DLD1 Caco-2	CRC tissues (n = 41)	***F. nucleatum* may modulate immune checkpoint therapy for CRC *via* activation of STING pathway.** Therapeutic responses of PD-1 blockade were positively associated with the level of ***F. nucleatum*** in CRC. ***F. nucleatum*** induced PD-L1 expression by activating STING signaling and increased the accumulation of IFNγ^+^CD8^+^ TILs during treatment with PD-L1 blockade, thereby enhancing anti-tumor effect and therapeutic responses of PD-L1 blockade in mice and prolonged survival. The therapeutic response of PD-L1 blockade in CRC organoids was improved by *F. nucleatum*.	(119)
**CRC**	MC38 tumor-bearing model	*Ptpn11^Lyz2^* ^-/-^	THP1	RNA-seq dataset from 23 patients	**SHP2 is a promising target for colon cancer immunotherapy.** Negative correlation of *PTPN11* and STING mediated type I IFN signaling was found in CRC tissues. STING signaling was negatively modulated by SHP2 (encoded by *PTPN11*) *in vitro*. SHP2 allosteric inhibitor **SHP099** restricted cancer malignant evolution by inducing T cell activation and type I IFN signaling response in a STING-dependent way.	(120)
**CRC**	-	*-*	-	CRC tissue and adjacent tissues (n = 87)	**PD-L1 might promote the occurrence of disease and STING might play an important role in anti-tumor immunity.** STING was significantly decreased in CRC tissues compared to corresponding adjacent tissue. Survival rates of CRC patients were higher in patients with STING positive expression and PD-L1 negative expression.	(121)
**CRC**	MC38 and CT26 tumor-bearing model	–	–	–	**Tumoral STING contributed significantly to STING-induced anti-tumor immunity, and combining epigenetic drugs, e.g., KDM5 inhibitors, may achieve better clinical response.** Loss of STING in tumor cells had no impact on the proliferation *in vitro* but significantly accelerated tumor growth in mice. KDM5 inhibitor **CPI-48** induced STING expression in CRC cells and suppressed tumor growth in immunocompetent mice in a STING-dependent manner.	(122)
**CRC**	CT26 tumor-bearing model	*Sting^gt/gt^*	-	-	**STING-triggered tumor-migrating macrophages participated in the anti-tumor effects of STING-activating compounds**. Intratumoral injections of **cGAMP** significantly delayed tumor growth in CD8^+^ T cells and STING signaling-dependent way. CD45^+^CD11b^mid^ Ly6C^+^MHC class II^+^ macrophages that produced TNF-α and T cell-recruiting chemokines transiently accumulated in mouse tumor microenvironment after intertumoral injection of cGAMP to perform anti-tumor effect.	(123)
**CRC**	MC38 tumor-bearing model	–	–	CRC tissues (n =225)	**STING could be a potential therapeutic target that enhances anti-cancer immune response in CRC.** Higher STING expression in CRC tissues was correlated with higher recurrence free and survival rates. The growth of tumor can be effectively suppressed after intratumoral STING agonist **3'3'-cGAMP** by enhancing intratumoral infiltration and activation CD8^+^ T cells.	(124)
**CRC**	MC38 and CT26 tumor-bearing model	*Sting^gt/gt^*	-	-	**diABZI combined with 1-MT could be a promising option for CRC.** **diABZI** combined with indoleamine 2,3 dioxygenase inhibitor 1-MT induced tumor regression, promoted the recruitment of CD8^+^ T cells and DCs, limited the infiltration of myeloid-derived suppressor cells, prolonged the survival time of CRC mice in a STING-dependent manner.	(125)

In addition, the efficacy of anti-tumor therapies is dependent on the cGAS-STING signaling. Evidence has shown that higher STING expression in human CRC specimens was associated with better survival and responsiveness to chemotherapy 5‐Fluorouracil (5‐FU) (112), a widely used chemotherapeutic drug, whose responsiveness requires a fully functional cancer cell-intrinsic cGAS-STING pathway, as well as IFN‐sensing by bone‐marrow‐derived cells (BMDCs) (112). Also, cGAS-STING-mediated type I IFN production in the host immune system was required for radiotherapy-induced adaptive immune responses (128) and anti-signal regulatory protein α (SIRPα)/anti-programmed cell death protein 1 (PD-1)/radiotherapy treatment (118). Additionally, it suggested that the capability of Midostaurin (PKC412) to restrict colorectal carcinoma cell growth is associated with the activation of the cGAS-STING signaling (113). Src homology-2-containing protein tyrosine phosphatase 2 (SHP2), encoded by the *PTPN11* gene, plays a critical role in the tumor microenvironment by regulating T cell activation, as well as polarization, phagocytosis, anti-inflammatory cytokine expression of macrophages (129). CRC patients with microsatellite stable phenotypes displayed increased CD68^+^ macrophage infiltration and enhanced SHP2 phosphorylation than microsatellite instability-high phenotypes, suggesting inhibition of SHP2 could be a promising strategy for CRC therapy. As an SHP2 allosteric inhibitor, SHP099 was shown to arrest the malignant evolution of tumor cells by activating STING-TBK1-IRF3-mediated type I IFN signaling in macrophages (120). In colon cancer cells, SHP2 was indicated to dephosphorylate poly (ADP-ribose) polymerase 1 (PARP-1) and thus to suppress DNA repair while promoting the cGAS-STING activation to elevate the sensitization of chemotherapy in colon cancer cells (111, 130). Although different mechanisms of SHP2-mediated STING activation in cancer cells and immune cells were suggested, the cGAS-STING signaling appears to be critically involved in anti-cancer immunotherapy.

As DNA and CDNs from bacteria are triggers of the cGAS-STING pathway, the regulatory roles of gut microbiota on the effects of STING signaling during the development of CRC also gained attention. AOM/DSS-treated *Sting* ^-/-^ mice housed under *Helico^+^* barrier facility showed significant bacterial differences in *Turicibacter* and *Odoribacter* species compared to that of healthy WT mice and antibiotic treatment eliminated the observed colitis and CAC in this model compared to untreated *Sting* ^-/-^ mice (110). On the other hand, no obvious changes in *Prevatollaceae*, *Bacteroides*, mouse intestinal *Bacteroides* (MIB), segmented filamentous bacteria (SFB) and TM7 were observed between *Sting^gt/gt^* and WT mice housed in an SPF facility (109). Therefore, the role of STING in CAC development might be dependent on the presence of specific commensal bacteria and opportunistic pathogens. Also, the regulatory role of intestinal microbiota that influences the efficacy of immunotherapies in a STING-dependent manner has been observed. Cluster of differentiation 47 (CD47), a ligand of SIRPα, is an immunoglobulin-like transmembrane protein that is elevated in many cancer cells (131–134), including colon cancer cells (135–137). The recognition of tumors presenting CD47 macrophages and dendritic cells is impaired, therefore, anti-CD47 immunotherapy is a potential therapeutic target of cancer treatment (138, 139). Administration of a *Bifidobacterium* cocktail (*B. bifidum*, *B. longum*, *B. lactis*, *and B. breve*) or STING agonist DMXAA synergized the anti-tumor efficacy of CD47 blockade in mice non-responders in a STING-dependent way. The potential of *Bifidobacterium* to enhance anti-tumor efficacy might be dependent on type I IFN signaling in DC and CD8^+^ T cell activation (114). In addition to anti-CD47 immunotherapy, bacteria can also increase the efficacy of anti-PD‐1/PD‐L1 therapy. Oral administration of probiotic *Lactobacillus rhamnosus* GG (LGG) augmented the anti-tumor responses to anti-PD-1 in colon cancer model in a cGAS-STING-dependent way (115). Moreover, tumor sensitivity to PD-1/PD-L1 checkpoint blockade can be enhanced by *F. nucleatum* supplementation, which induced PD-L1 expression by activating STING signaling and increasing the abundance of IFN-γ^+^CD8^+^ tumor-infiltrating lymphocytes in the tumor tissues of BALB/c mice during treatment with PD-L1 blockade (119). Since the application of PD-L1 blockade in CRC was not effective in clinical trials (140, 141), exposure to specific microorganisms might be a promising strategy to improve the efficacy of cancer immunotherapies. It is worth noting that although supplementation of certain bacteria can synergize immunotherapies, bacteria such as *F. nucleatum*, have been reported to be positively associated with the recurrence of CRC after chemotherapy by promoting chemoresistance, as well as increasing proliferation and invasive activities of CRC cell lines *via* activation of TLR4 and MYD88 signaling (142, 143). This suggests that STING signaling could have opposing roles in cancer immunity compared to other PRRs involved in microbiota recognition, and thus thoughtful investigation is required when using bacteria as adjuncts to activate STING-dependent immune response in CRC treatment. Therefore, more investigations are needed on how specific bacteria or their products can stimulate the cGAS-STING pathway and enhance the anti-tumor immune response, which may promote the development of novel therapeutic strategies or interventions for CRC treatment. In addition, applying STING agonists alone or combined with traditional CRC immunotherapies is also a promising strategy to suppress tumorigenesis. Treatment with the STING agonist DMXAA in a murine CAC model reduced structural heteromorphism, the number of adenocarcinomas and increased immune cell infiltration. Increased Syk-mediated pyroptosis in tumor cells was found in an *ex vivo* study (108). After intertumoral injection of cGAMP, a macrophage phenotype-like cell subset that produced TNF-α, displayed phagocytic activity and expressed high levels of T-cell recruiting chemokines, were transiently accumulated in the tumor microenvironment of CRC mice model (123). Also, the STING agonist diABZI combined with the indoleamine 2,3 dioxygenase (IDO) inhibitor 1-MT significantly inhibited tumor growth and promoted the recruitment and activation of IFN-γ^+^CD8^+^ T cells and DCs, and significantly decreased myeloid-derived suppressor cells that exerted immunosuppressive function (125). STING agonist (cGAMP or RR-CDA) treatment combined with VEGFR2 blockade (DC101) can induce tumor regression in the CT26 colon cancer mouse model, which was largely dependent on IFNAR and CD8^+^ T cells (116). Intraperitoneal application of the STING agonist RR-CDA (also known as MIW815 or ADU-S100) alone or combined with an anti-PD-1 antibody enhanced antitumor immunity and normalized aberrant angiogenesis in peritoneal carcinomatosis of colon cancer (144).

Thus, applying STING agonists or bacteria that can enhance the cGAS-STING signaling in combination with traditional anti-tumoral therapies could be a strategy for CRC treatment in the future. Recently, phase I clinical trials with the STING agonists MIW815 (ADU-S100) and SYNB1891 (an engineered *Escherichia coli* Nissle strain 1917 expressing STING agonist CDNs under hypoxia) as monotherapy or in combination with immune checkpoint inhibitors (anti-PD1 or anti-PD-L1) have been conducted in patients with solid tumors (including CRC), lymphomas or advanced/metastatic cancers (145–147). These clinical trials have shown immune response activation and good tolerance in patients, but the anti-tumor effect was limited. Moreover, there are registered clinical studies in phase I and phase II using STING agonists, such as SNX281, TAK-500 and IMSA101 in patients with solid tumors or lymphomas (ClinicalTrials.gov identifiers: NCT04609579; NCT05070247; NCT05846659). Other clinical trials supporting the use of STING agonists in cancer immunotherapy have recently been reviewed and discussed by Hines et al. (148). These investigations will contribute to a better understanding of the cGAS-STING pathway activation as a therapeutic target in cancer. However, advances in basic and clinical research on optimal therapeutic dose, delivery system, and possibly drug combinations are needed to improve the anti-tumor effects of STING agonists to achieve an effective and safe therapy. Further understanding the interaction of cGAS-STING signaling, gut bacteria and the tumor microenvironment is critical to evaluate the possibility of targeting the cGAS-STING pathway in clinical implications.

## The cGAS-STING signaling pathway in intestinal inflammatory bowel diseases

Inflammatory bowel diseases (IBD), including ulcerative colitis (UC) and Crohn’s disease (CD), are long-lasting, relapsing-remitting, and chronic inflammatory diseases of the intestinal tract. The complex multifactorial etiology of IBD poses a great challenge to prevent disease progression and develop effective therapeutic strategies (149, 150). Multiple studies have suggested the pro-inflammatory role of cell-free DNA in IBD. Plasma mtDNA levels were increased in the DSS-induced colitis mouse model as well as in both UC and CD patients, in which mtDNA levels were positively correlated with disease severity (151–153). Increased DNA damage and accumulation of cytosolic DNA were also observed in the colon of colitis mice model and IBD patients (154, 155). Microbiota dysbiosis that influences the level of CDNs has been shown to be involved in the pathogenesis of IBD as well (156–158). Therefore, due to its capacity of sensing dsDNA and CDNs from bacteria, increasing research focus on exploring the role of the cGAS-STING pathway in IBD **(** **Table 2** **)**. Several studies reported that the cGAS-STING pathway is activated in UC patients with active disease. Protein levels of cGAS, p-STING, p-TBK1 and p-IRF3 were increased in the colon of UC patients with active disease compared to patients in remission and healthy controls (159). In addition, the expression of STING was increased in inflamed regions of the colon in UC patients compared with healthy control and noninflamed flanking areas (103). Although *STING*, *TBK1*, and *IRF3* mRNA levels were unchanged in a study by Chen et al. (159), Flood et al. reported a significant increase of *STING*, *TBK1*, *IRF3*, *IFNB1* gene levels in active UC, which supports that the STING-TBK1-IRF3 axis was activated in UC (165). Similarly, a significant increase of p-STING, p-IRF3 and p-p65 protein levels were detected in colon tissues of active CD patients (153). Autophagy related 16 like 1 (*ATG16L1*) is a genetic risk factor of CD (166). Mice with a deletion of *Atg16l1* in the IECs develop spontaneous ileitis phenocopying ileal CD (167). An *in vitro* study showed that cell death was significantly enhanced in *Atg16l1* ^ΔIEC^ organoids treated with IFN-β or IL-22 that activate STING signaling. Blocking the IFN receptor led to amelioration of the disease severity in mice (168). In addition, the stabilization of STING protein *via* bacteria-induced ubiquitination in mice challenged with colitis inducer *Salmonella* typhimurium, *Citrobacter rodentium* further exacerbated colitis in mice (103).

**Table 2:** Strategies targeting the cGAS-STING pathway in intestinal inflammatory diseases.

Disease type	Mice models	GM-mice lineages	Human Cell lines	Human clinical data	Main finding about the mechanisms and role of the cGAS-STING signaling and possible therapies/agents targeting the cGAS-STING pathway to improve disease progression/outcomes	Reference
**CD**	DSS-induced colitis	*Sting* ^-/-^	-	Colon sample from CD patients (n = 10) and non-CD control (n = 9).	**Exosomal dsDNA promoted intestinal inflammation via activating the STING pathway in macrophages**. Elevated dsDNA levels in EVs of plasma were found in active CD patients and DSS-induced mice. Phosphorylated STING and p-IRF3 proteins were increased in the colon tissues and macrophages of active CD patients. Also, EVs from the plasma of active human CD triggered STING activation in macrophages in vitro. Blockade of EVs by intraperitoneal injection of **GW4869** ameliorated murine colitis by inhibiting the activation of the STING pathway.	(153)
**UC**	DSS-induced colitis	*Sting* ^+/N153s^ *Ifnar* ^-/-^ *Tcrδ* ^-/-^ *Tcrβ* ^-/-^ *Rag1* ^-/-^	–	Affected and adjacent unaffected colon from UC patients (n = 3-5).	**Dysbiosis fomented the accumulation of STING in intestinal myeloid cells, driving intestinal inflammation.** STING expression was elevated in the affected colon of UC patients compared with unaffected regions of UC patients and healthy control. *Sting* ^+/N153s^ (N153S) mice displayed reduced body weight, shortened colon, server histopathology in the colon and intestinal fibrosis. Intestinal inflammation in N153S mice was associated with T-cell accumulation in the colon. STING accumulated mainly in myeloid cells under conditions of intestinal inflammation. Intrinsic activation of STING in myeloid cells drove intestinal inflammation.	(103)
**UC**	DSS-induced colitis	-	HT-29	Colon tissues from active UC (n = 15), remissive UC (n = 9) and the control (n = 45).	**ANP can be used as a pharmacologic inhibitor of STING signaling pathway, by targeting pro-inflammatory cytokines and modulating gut barrier dysfunction and ER stress-induced autophagy in UC.** *cGAS* mRNA, p-STING, p-TBK1, and p-IRF3 protein were increased in active UC compared to the remissive UC and the control group. Intraperitoneally injected with **ANP** ameliorated DSS-induced colitis was associated with the suppression of the STING pathway. The epithelial barrier damage and ER stress-induced autophagy caused by DMXAA *in vivo* were improved after ANP treatment.	(159)
**UC**	DSS-induced colitis	*Sting ^gt/gt^*	–	Affected and adjacent unaffected areas of colon from UC patients (n = 4).	**Ganciclovir (GCV) may be useful for the treatment of UC through inhibiting STING signaling in colonic macrophages.** STING in colon tissues was increased in the affected area of UC patients and the DSS-induced mice model. The mRNA levels of *cGAS* and *STING* were increased in CD and UC patients. Intraperitoneal injection of low-dose **GCV**, an antiviral drug, can ameliorate the symptoms of ulcerative colitis in mice, as evidenced by reduced weight loss, lower disease activity index, and decreased histological damage to the colon tissues, possibly through inhibiting STING activation in macrophages.	(160)
**UC**	DSS-induced colitis	-	-	-	**Si-Ni-San (SNS) may serve as a novel therapeutic strategy for the treatment of UC *via* interfering type I IFN-mediated inflammation.** SNS significantly reduced the severity of chronic colitis in mice, as evidenced by decreased body weight loss, reduced colon length shortening, and decreased histological damage to the colon tissues, which was associated with its role to inhibit type I IFN-related genes by regulating STING and RIG-I-dependent pathways.	(161)
**IBD**	DSS-induced colitis	–	–	–	**Prop-2-yn-1-one had potential to be further developed for inflammatory disease therapies.** Pretreatment of a novel cGAS inhibitor inhibited the activation of STING pathway after Interferon Stimulatory DNA treatment in macrophages via covalently bind to Cys419 of cGAS. Intraperitoneally injected cGAS inhibitor ameliorated DSS-induced colitis in mice.	(162)
**IBD**	*-*	*Atg16l1* ^ΔIEC^ *Xbp1* ^ΔIEC^ *Sting ^gt/gt^* *Cgas^-/-^*	*-*	-	**ATG16L1 and STING coordinated the outcome of IL-22 signaling in the intestinal epithelium.** IL-22 stimulation physiologically leaded to transient ER stress and subsequent activation of STING-dependent type I IFN signaling, which was enhanced in *Atg16l1* ^ΔIEC^ intestinal organoids. Type I IFN signaling amplified epithelial TNF production and led to necrotic cell death. *In vivo*, IL-22 treatment in *Atg16l1* ^ΔIEC^ and *Atg16l1* ^ΔIEC^/*Xbp1* ^ΔIEC^ mice enhanced endogenous ileal inflammation and resulted in widespread necrotic epithelial cell death. Therapeutic blockade of type I IFN signaling ameliorated IL-22-induced ileal inflammation in *Atg16l1* ^ΔIEC^ mice.	(163)
**IBD**	–	*Sting* ^-/-^		Inflamed colon tissues from IBD patients. (n = 5-7)	**STING played a critical role in mediating inflammation response during acute colitis.** The protein level of STING was increased in UC and CD patients. STING deficiency alleviated acute experimental colitis in mice, which was associated with the decrease of infiltration of CD45^+^ leukocyte, CD3^+^ T cell, F4/80^+^ macrophage and MPO^+^ neutrophil in colonic tissues and the expression of pro-inflammatory cytokines.	(108)
**IBD**	DSS-induced colitis	-	-	-	**cGAS protected the integrity of intestinal epithelial barrier.** Increased inflammation, ulceration and crypt distortion in colon of *Cgas-/-* mouse. *Cgas* ^−/−^ but not *Sting* ^gt/gt^ mice exhibited impaired gut barrier function as evidenced by the leakage of FITC-dextran from the gut lumen to the blood.	(107)
**IBD**	DSS-induced colitis	*GSDMD* ^-/-^			**GSDMD protected against colitis through restricting cGAS-dependent inflammation.** Cytosolic DNA-sensing pathway was upregulated in CD11b^+^F4/80^+^ cells in *Gsdmd* ^-/-^ mice, in which many genes were associated with cGAS, companied with higher levels of cGAMP in colon. Phosphorated-STING, p-TBK1 and p-IRF3 were increased in in the *Gsdmd* ^−/−^ colonic tissues compared with controls after DSS challenge. The intraperitoneal injection of cGAS inhibitor **RU.521** alleviated intestinal colitis in *Gsdmd* ^−/−^ mice.	(164)
**IBD**	DSS/T-cell *Salmonella* typhimurium induced-colitis	*Sting* ^-/-^	-	-	**STING maintained gut homeostasis and showed protective effect in controlling gut inflammation**. *Sting-/-* mice showed the overgrowth of bacteria that was associated with gut inflammation. *Sting* ^-/-^ mice had a higher susceptibility to DSS-induced, T-cell induced and *S*. typhimurium-indued colitis.	(101)

In contrast to the protective role of the cGAS-STING pathway in CRC cancer, the cGAS-STING pathway appears to promote inflammation and intestinal barrier dysfunction in IBD **(** **Figure 3C** **)**. For instance, extracellular vesicles (EVs) carrying dsDNA from the plasma of active CD patients and damaged CT26 cells triggered STING activation in bone marrow-derived macrophages (BMDMs) *in vitro* and promoted the expression of pro-inflammatory cytokines, while this was not observed in *Sting*-deficient macrophages. And the level of intestinal apoptosis and pro-inflammatory cytokines were reduced in *Sting*-deficient mice compared with DSS-treated wild-type mice (153). The detrimental effects of overactivated STING signaling in IBD have also been investigated in the N153S mice model in which STING is constitutively activated. N153S mice spontaneously develop severe colitis and intestinal fibrosis with barrier dysfunction, which could be associated with the predominant presence of active STING in myeloid cells (103). It is worth noting that STING gain-of-function mutation in human causes STING-associated vasculopathy with onset in infancy (SAVI), a rare disease associated with systemic inflammation, resulting in peripheral vasculopathy, interstitial lung disease, polyarthritis and respiratory failure (169, 170). Unlike its mice model, the gastrointestinal involvement in SAVI has not been addressed. Although SAVI has distinct underlying mechanisms and clinical presentations compared with UC, there is a connection between them in terms of the potential use of JAK inhibitors for treatment (171). In apparent agreement, *Sting* ^-/-^ mice appear resistant to DSS treatment, exhibiting less weight loss, disease activity, and longer colon length than DSS-treated WT controls (108). Treatment with the STING agonist DMXAA significantly decreased expression of the tight junction proteins (TJPs) ZO-1 and occludin in human colorectal adenocarcinoma cell HT-29 and in the DSS-induced colitis mouse model when compared to DSS treatment alone (159). *Il-10* deficiency is a genetic mouse model that causes chronic enterocolitis (172), and *Sting* deficiency in *Il-10* ^-/-^ mice ameliorated the severity of inflammation *via* reducing pro-inflammatory cytokines expression (110). Atrial Natriuretic Peptide (ANP) treatment displayed its ability to restrict ER stress-induced autophagy and restore TJPs level *via* restricting the cGAS-STING pathway in colon tissues of DSS-induced mouse model (159). Unlike *Sting* deficiency, the absence of *Cgas* worsened colitis and decreased autophagy in the DSS-induced mouse model independent of microbiota (173), although cGAS inhibitor illustrated benefit in restricting inflammation in DSS-induced colitis mouse model (162).

Overall, the above results suggest that gut microbiota is involved in the modulation of the cGAS-STING pathway in the context of inflammation. Lowering the production of cell-free DNA and CDNs, restricting the cGAS-STING as well as regulating intestinal flora might be potentially helpful in modulating inflammation and barrier dysfunction in IBD. Still, while inhibiting the overactivation of STING signaling might suppress intestinal inflammatory immune response and IBD progression, total blockade of the cGAS-STING activity may also induce intestinal pathologies, as suggested by studies with *Sting* ^-/-^ mice.

## The cGAS-STING signaling in intestinal ischemia-reperfusion and sepsis

Intestinal ischemia/reperfusion (I/R) injury is caused by an insufficient blood supply (ischemia) followed by a restoration of blood flow (reperfusion) in the intestinal tissue, as during septic shock, severe trauma and gastrointestinal surgeries like intestinal resection or small intestine transplantation. Intestinal I/R injury leads to an increase in oxidative stress, cell death, and epithelial barrier breakdown with the consequent release of intracellular components, known as DAMPs. Bacteria translocation from the lumen to the lamina propria can result in intestinal complications, affecting distant organs (e.g. acute liver injury) or leading to a systemic inflammatory response syndrome (SIRS) or multiple organ dysfunction syndrome (174, 175). As a DAMP and also ligand of STING signaling, mtDNA released from damaged and stressed mitochondria were increased in patients after gastrointestinal surgery with SIRS (176), in a mouse and cell model of intestinal I/R (177), and in cecal ligation perforation (CLP)-induced sepsis in mice **(** **Table 3** **)** (178). Furthermore, the expression of STING and its downstream effectors p-IRF3, NF-κB, inflammatory cytokines (TNF-α, IL-1β, IL-6), apoptosis and epithelial injury were increased in intestinal tissues from patients with abdominal sepsis (178). While *Sting* deficiency or DNAse I treatment mitigated CLP-induced inflammation, IEC apoptosis and intestinal barrier damage in mice (178). Further research indicated that mtDNA can promote necroptosis signaling in IECs and therefore exacerbate intestinal injury in WT mice. Compared with WT mice, *Sting* ^-/-^ mice exhibited improved intestinal morphology and reduced intestinal inflammation and necroptosis after the intraperitoneal injection of mtDNA (181). Moreover, the absence of *Sting* ameliorated damage to liver and lung as well as lipid peroxidation in intestinal I/R mice model (179) **(** **Figure 3D** **)**. The STING antagonist H151 was also shown to ameliorate lung and intestinal injury, as well as systemic inflammation after intestinal I/R (182). However, it remains unclear which types of cells are responsible for the therapeutic effects of H151. These results suggest that the combination of oxidative stress inhibitors (antioxidants), STING antagonists, and the elimination of mtDNA could be a potential therapeutic strategy for restoring intestinal barrier disruption and therefore to improve intestinal I/R injury.

**Table 3:** Strategies targeting the cGAS-STING pathway in sepsis and intestinal I/R injury.

Disease type	Mice models	GM-mice lineages	Human Cell lines	Human clinical data	Main finding about the mechanisms and role of cGAS-STING signaling and possible therapies/agents targeting cGAS-STING pathway to improve disease progression/outcomes	Reference
**Sepsis**	CLP-induced sepsis	*Sting* ^-/-^	-	Intestinal biopsies from abdominal sepsis patients (n = 5)	**Targeting the mtDNA-STING pathway could potentially serve as a therapeutic approach to mitigate intestinal barrier disruption and promote mucosal healing in septic patients.** STING pathway was activated in human sepsis and CLP-mouse model. *Sting* deficiency ameliorated multi-organs (lung, liver, and kidney) injury and intestinal inflammation, IEC apoptosis and intestinal barrier dysfunction, while *STING* agonist DMXAA following CLP procedure amplified disease pathogenesis in mice. Intraperitoneal injection **DNase I** inhibited intestinal damage and bacterial translocation.	(178)
**Intestinal I/R injury**	I = 45 min R = up to 2 h	*Sting* ^-/-^	–	–	**STING inhibition mitigated lipid peroxidation-associated intestinal I/R injury.** STING deficiency ameliorated intestinal and distant organs (lung and liver) damages, reduced the levels of plasma inflammatory cytokines and lipid peroxidation. Malondialdehyde of BMDMs from *Sting* ^-/-^ mice showed less induction upon mtDNA stimulation. DMXAA-induced macrophage death in a dose- and time-dependent way can be reversed by pretreatment of **Liproxstatin-1**.	(179)
**Intestinal I/R injury**	I = 60 min R = up to 3 h	*Sting* ^-/-^	-	-	**STING inhibition improved acute lung injury caused by intestinal I/R might be associated with AMPK signaling.** Intraperitoneal injection of STING inhibitor **C-176** mitigated lung injury and pulmonary fibrosis, cell death (pyroptosis and apoptosis) induced by intestinal I/R, which was associated with the increased level of p-AMPK.	(180)
**Intestinal I/R injury**	I = 45 min R = up to 2 h	*Sting* ^-/-^	–	–	**mtDNA-STING signaling contributed to intestinal I/R injury by promoting IEC necroptosis.** *Sting* deficiency alleviated intestinal injury, barrier damages, necroptosis and the increase of pro-inflammatory cytokines. Intraperitoneal injection of necroptosis inhibitor **Necrostatin-1** before the induction of I/R alleviated intestinal I/R-induced damages, decreased histological score and the plasma levels of pro-inflammatory cytokines.	(181)
**Intestinal I/R injury**	I = 60 min R = up to 4 h	*-*	-	-	**STING inhibition ameliorated lung and intestine injury and improved survival after I/R injury** STING antagonist **H151** restricted extracellular cold-inducible RNA-binding protein (eCIRP)-induced activation of STING *in vitro*. H151 treatment reduced organ injury markers (LDH and AST), pro-inflammatory cytokines (IL-1β and IL-6) and tissue injury in the I/R injury mice model. H151 treatment restricted neutrophil infiltration and chemokine expression in the intestine and lung and improved survival outcomes.	(182)

Taken together, the involvement of the STING pathway in intestinal damage reveals a dual function depending on the causes underlying the intestinal disturbance. On the one hand, the STING pathway triggered by released mtDNA following intestinal I/R injury causes a local and/or systemic inflammatory response. On the other hand, as we mentioned before, the immune response generated by the STING pathway following acute damage caused by irradiation led to the resolution of inflammation and epithelial regeneration (104). Further investigation into the mechanisms under different types of acute or chronic intestinal injury will clarify when interventions with DAMPs inhibitors, STING agonists, or antagonists are most effective.

## STING in intestinal parasite and viral infections

Intestinal parasite infections have a high morbidity and mortality rate in endemic countries and are the most common and prevalent infections worldwide (183, 184). Parasite infections can cause intestinal symptoms, such as diarrhea, abdominal pain, inflammation and bleeding, which vary depending on the specific parasite involved and the severity of the infection. Many reports have shown that the cGAS-STING signaling can be triggered by parasites including *Plasmodium* (185), *Toxoplasma gondii* (186), *Leishmania* (187), *Trypanosoma* (188), *Schistosoma* (189), while the role of the cGAS-STING pathway in parasite infections remains controversial. Wang et al. found that cGAS was required for the activation of the immune signaling defense against *Toxoplasma gondii* in a mouse model (186). Also, STING agonist c-di-AMP could protect against *Trypanosoma cruzi* infection by promoting Th1/2/17 cells and CD8^+^ T cells responses (188). However, although *S. japonicum* and *S. mansoni* can activate cGAS-STING-dependent type I IFN response, and surprisingly, *Cgas^-/-^* mice and *Sting^-/-^* mice were more resistant to *S. japonicum* and *S. mansoni* infection (189, 190). The resistance of *Cgas* or *Sting* deficiency to parasite infection has also been observed in Malaria infections caused by *Plasmodium*. *Plasmodium yoelii* and *Plasmodium falciparum* malaria infection can cause intestinal pathological changes, such as intestinal shortening, intestinal barrier damage, increased mucus thickness and gut dysbiosis (191, 192). Mice deficient in *Cgas*, *Sting*, or *Ifnar* were resistant to *Plasmodium yoelii* infection (193, 194). As shown by Du et al. (195), the immune response in *Cgas* or *Sting*-deficient mice resistant to lethal malaria strains *Plasmodium yoelii* N67C and YM infection was characterized by an elevation of type I IFN in the early stages of infection and a reduction of IL-6 in the late stages. In the context of cGAS-STING activation, late IL-6 production was promoted in macrophages *via* MyD88-p38 signaling, resulting in the expansion of proinflammatory monocytes (CD11b^+^Ly6C^hi^) and the suppression of T cell proliferation, resulting in loss of the immune defense against malaria. Therefore, more studies are required to illustrate the role of the cGAS-STING pathway in intestinal mucosa in the context of specific parasite infections for developing targeted therapeutic interventions.

Rotavirus, norovirus, adenovirus, and astroviruses are the most common causes of acute viral gastroenteritis worldwide, presenting symptoms such as vomiting, diarrhea, nausea and abdominal pain (196, 197), where the STING pathway protects against viral infections (27, 198–200). In addition, although Human Herpesviruses (HHV) were rarely found in the gastrointestinal tract in adults (201, 202), it is positively associated with morbidity and mortality in patients with IBD, especially in immunocompromised individuals (203). Moreover, persistent infection of the intestinal epithelial cells and systemic dysfunction was observed in murine gammaherpesvirus-68 (γHV-68)-exposed mice, a model to understand the pathogenicity of HHV-8 (204). It was reported that HHV such as Herpes simplex virus-1 (HSV-1) can infect enteric neurons and recruit activated CD3^+^CD8^+^ lymphocytes, resulting in gastrointestinal neuromuscular dysfunction in mice model (205). *Sting* ^-/-^ and *Cgas* ^-/-^ mice appeared highly susceptible to HSV-1 infection. Also, the STING pathway seemed to enhance the adjuvant activity of DNA-vaccine-induced T cell activation and antibody production in response to antigen (49, 206). The fact that Hepatitis B virus (HBV) and Hepatitis C virus (HCV) led to the compositional and functional alterations in the gut microbiota, which further impairs the intestinal barrier and nutrition absorption, has been considered to promote the progression of hepatitis (207, 208). Although it has been shown that STING activation-mediated autophagic flux inhibited inflammasome activation in macrophages, ameliorated liver injury and fibrosis in mouse model induced by HBV (209), it remains to be explored that whether STING pathway-mediated attenuation of hepatitis is associated with restoration of gut dysbiosis. Furthermore, it is well known that influenza virus causes infectious respiratory disease with common symptoms such as cough, fever, headache, while it also impaired gut barrier and immunity, leading to gastroenteritis-like symptoms (210, 211). The STING-dependent antiviral response is essential for limiting virus propagation (212, 213). The STING pathway also contributed to IFN response to the severe acute respiratory syndrome coronavirus 2 (SARS-CoV-2/COVID-19) (214, 215), causing intestinal ischemia and thrombosis (216, 217), mesenteric vascular endothelial cells damages (218) and increased risk of post-acute gastrointestinal pathologies (219). These data illustrate that the STING pathway is necessary for host defense against viral infections and understanding how STING signaling responds to viral infection might be important in the development of anti-viral therapies.

It is worth noting that viruses have developed multiple mechanisms to fight the host IFN responses that are controlled by STING. The HSV-1 ICP27 protein interacted with STING and TBK1 to inhibit the expression of type I IFN in human macrophages (220). HSV-1 can also evade the antiviral immune response by inducing miR-24 that binds to the 3’untranslated region of the STING mRNA and inhibit its translation (221). Human cytomegalovirus (HCMV), another virus that belongs to Herpesviridae, whose tegument protein pUL83 (also known as pp65) and UL82 (also known as pp71) can suppress the immune response by interacting with the IFI16 pyrin domain (222) and impair the trafficking of STING from the ER to perinuclear microsomes as well as the recruitment of TBK1 and IRF3 to the STING complex (223), thereby promoting HCMV replication and immune evasion. Additionally, the NS4B protein of HCV can disrupt the interactions between STING and TBK1 and thereby block the host interferon signaling responses (224). Influenza virus is able to increase cytosolic levels of mtDNA and cGAMP, leading to stimulation of cGAS-STING-dependent IFN-β expression. The NS1 protein of influenza virus was associated with mtDNA through its RNA-binding domain to evade the STING-dependent antiviral immunity (213). Therefore, targeting viral proteins involved in virus infection while combining with enhancing STING signaling might be a more efficient antiviral strategy. Controversially, a recent study showed that STING can facilitate nuclear import of HCMV and HSV-1 dsDNA during infection, rendering originally HCMV-insusceptible cells susceptible to the virus. The reduction of HSV-1 gene expression and HSV-1 viral DNA in the nuclear fraction were confirmed in *STING* ^-/-^ HepG2 cells as well. STING deficiency negatively regulated the establishment of HCMV latency and reactivation in monocytes, the latent reservoirs of HCMV (225), suggesting that STING might have a proviral effect in DNA virus infection and more investigations in different types of cells are required. Overall, the STING pathway is essential in DNA and RNA virus defense and could be a target for antiviral therapies. Although virus infections frequently cause intestinal manifestation, the cellular mechanism of the cGAS-STING pathway in intestinal mucosa during virus infection needs more research.

## Conclusion and future perspectives

Ever since the initial discoveries that the cytosolic DNA sensor cGAS produces cGAMP to activate STING (20, 226), the cGAS-STING pathway has gained increased attention due to its critical role in regulating immune responses and has emerged as a promising therapeutic target in intestinal diseases. Detailed analysis of this pathway led us to better understand the host immune system in response to genetic susceptibility and environmental complexity. In this review, we focused on the role of the cGAS-STING pathway in intestinal homeostasis and diseases, which included their role in normal gut physiology, CRC, IBD, intestinal IR injury and intestinal infections caused by viruses, bacteria and parasites. Data so far show that the cGAS-STING pathway exhibits opposite effects in the progression of IBD and colon cancer. In IBD, activation of the cGAS-STING pathway promotes the production of pro-inflammatory cytokines and contributes to inflammation in the gut, while in colon cancer, its role skews to recruit immune cells to the tumor microenvironment and suppress tumor growth. These distinct effects might involve many factors, such as genetics, microbiota, tumor microenvironment and immune context. Precisely modulating the cGAS-STING pathway in cancer therapeutic interventions could be crucial to limit its potential adverse effects. Similarly, restricting overactivated STING signaling seems to be a promising therapeutic target for IBD, but likely some basal level of STING activation needs to remain to maintain gut homeostasis. Despite the growing therapeutic interest in this signaling cascade, several limitations hinder a comprehensive understanding of this pathway. Firstly, the majority of studies have primarily focused on immune cells, such as macrophages, dendritic cells, NK cells and T cells, while the influence of the cGAS-STING pathway in other cells, including epithelial cells and stromal cells, remains relatively unexplored. Expanding investigations to non-immune cells in intestinal diseases might be helpful in understanding the intricate interaction and communication of cells and provide insight into developing cell-targeted therapies and interventions. Secondly, it is known that there are several DNA sensors that play crucial roles in detecting and responding to different types of DNA in the host immune system and they are also involved in the progress of diseases, such as autoimmune diseases and cancer (227). Analyzing the crosstalk between the cGAS-STING pathway and other DNA sensors might provide key insights into manipulating host immune responses. Also, a thorough investigation of the potential synergistic or antagonistic interplay between cGAS-STING and other PRRs could offer novel strategies for modulating intestinal disorders. Thirdly, we need better mechanistic understanding of the regulatory effects of post-translational modifications of STING. SUMOylation, ubiquitination, phosphorylation, and palmitoylation affect oligomerization and activation of STING (228–231), while dephosphorylation, deubiquitination, carbonylation, deSUMOylation may inhibit the dimerization, decrease the oligomerization or inhibit the residence of STING (230, 232–234). Deeper understanding of these post-translational modifications in intestinal diseases might provide novel perspectives in drug development.

## Author contributions

YY contributed to the manuscript concept and structure. YY, LW, IP-G and DP-V each wrote and contributed to sections of literature search, manuscript drafts, figures drafts and review. KF and GD contributed to the correction and supervision of the manuscript. All authors contributed to the article and approved the submitted version.
